# Xuefu Zhuyu decoction, a traditional Chinese medicine, provides neuroprotection in a rat model of traumatic brain injury via an anti-inflammatory pathway

**DOI:** 10.1038/srep20040

**Published:** 2016-01-28

**Authors:** Zhihua Xing, Zian Xia, Weijun Peng, Jun Li, Chunhu Zhang, Chunyan Fu, Tao Tang, Jiekun Luo, Yong Zou, Rong Fan, Weiping Liu, Xingui Xiong, Wei Huang, Chenxia Sheng, Pingping Gan, Yang Wang

**Affiliations:** 1 Laboratory of Ethnopharmacology, Institute of Integrated Traditional Chinese and Western Medicine, Xiangya Hospital, Central South University, 410008 Changsha, China; 2 Department of traditional Chinese medicine, 2nd Xiangya Hospital, Central South University, 410011 Changsha, China; 3 Thyroid Tumour Internal Medicine Department, Cancer Hospital affiliated to Xiangya School of Medicine, Central South University, 410013 Changsha, China; 4 Department of Pharmacy, Shaoyang Medical College Level Specialty School, 422000 Shaoyang, China; 5 Department of Gerontology and Respiratory Diseases, Xiangya Hospital, Central South University, 410008 Changsha, China; 6 Department of Oncology, Xiangya Hospital, Central South University, 410008 Changsha, China

## Abstract

Neuroinflammation is central to the pathology of traumatic brain injury (TBI). Xuefu Zhuyu decoction (XFZY) is an effective traditional Chinese medicine to treat TBI. To elucidate its potential molecular mechanism, this study aimed to demonstrate that XFZY functions as an anti-inflammatory agent by inhibiting the PI3K-AKT-mTOR pathway. Sprague-Dawley rats were exposed to controlled cortical impact to produce a neuroinflammatory response. The treatment groups received XFZY (9 g/kg and 18 g/kg), Vehicle group and Sham group were gavaged with equal volumes of saline. The modified neurologic severity score (mNSS) and the Morris water maze test were used to assess neurological deficits. Arachidonic acid (AA) levels in brain tissue were measured using tandem gas chromatography-mass spectrometry. TNF-α and IL-1β levels in injured ipsilateral brain tissue were detected by ELISA. AKT and mTOR expression were measured by western blot analysis. The results indicated that XFZY significantly enhanced spatial memory acquisition. XFZY (especially at a dose of 9 g/kg) markedly reduced the mNSS and levels of AA, TNF-α and IL-1β. Significant downregulation of AKT/mTOR/p70S6K proteins in brain tissues was observed after the administration of XFZY (especially at a dose of 9 g/kg). XFZY may be a promising therapeutic strategy for reducing inflammation in TBI.

Traumatic brain injury (TBI), an intracranial injury caused by an external force that exceeds the protective capacity of the brain, is the leading cause of mortality and disability in people under the age of 45 years^1^. Annually, 5.3 million people suffer from TBI in the United States^2^. Of these, 1.4 million people require emergency treatment and more than 235,000 require hospitalization^3^. In China, the proportion of people with severe TBI caused primarily by traffic accidents or high-level falls is much higher than that in other countries^4^. It is estimated that around 10 million people are affected by TBI worldwide annually^5^. Although several TBI therapies, such as progesterone^6^, minocycline, melatonin, statins and mesenchymal stem cells^3^,^7^, have shown promising results in basic research and early clinical trials, to date, none has succeeded in phase III clinical trials^8^.

The complex pathogenesis of TBI is initially induced by mechanical damage, which is followed by a series of secondary injury cascades^9^. During the acute and chronic stages of TBI, neuroinflammation has been implicated as the key to disease pathology and treatment^3^,^10^,^11^. Neuroinflammation is a robust, sterile immune reaction that is mediated by central nervous system (CNS)-resident and peripherally recruited inflammatory cells^12^. Following TBI, the burst of reactive oxygen species (ROS) and increased glutamate levels contribute to an inflammatory reaction^13^,^14^, which exacerbates the edema by increasing blood-brain barrier permeability^15^. In addition, inflammation prevents nerve regeneration by influencing glial scar formation^11^,^16^. Furthermore, the pathological process impairs cognitive memory by disrupting the macromolecular synthesis that is required for synaptic plasticity^17^. The inflammation that is induced by cerebral contusions via pathophysiological mechanisms causes 60% of the secondary damage^18^. Hence, treatments that target neuroinflammation have attracted much attention as possible treatments for TBI.

Arachidonic acid (AA (20:4ω6)) is a precursor of prostaglandins (PGs) and leukotrienes (LTs), both of which induce inflammatory cellular infiltration^19^. The first responders during inflammation are polymorphonuclear leukocytes (i.e., neutrophils), followed by monocytes, multipotent bone marrow-derived leukocytes that differentiate into macrophages^20^. These inflammatory cells that are recruited to the area of the lesion secrete several inflammatory factors^10^,^12^. TNF-α is the central cytokine that initiates and regulates the inflammatory response^21^. IL-1β plays an important role in the development and progression of the cellular inflammatory cascade^22^. In addition, microglia/macrophages that are present in brain tissue after trauma are activated and persist as the M1 phenotype, which primarily produce pro-inflammatory cytokines (e.g., TNF-α and IL-1β) during the chronic phase^10^. Subsequently, it is reasonable to consider AA, IL-1β and TNF-α as targets for assessing the degree of inflammation.

Mounting evidence indicates that the PI3K-AKT-mTOR signaling pathway has a critical role in fine-tuning the inflammatory response^23^,^24^,^25^. PI3K-AKT-mTOR is a pivotal regular pathway of cell growth and metabolism that integrates various environmental signals^8^,^26^. At the onset of brain trauma, the AKT-mTOR signaling pathway is activated in the brain parenchyma owing to the irritable brain status of TBI^27^,^28^,^29^. AKT is mainly activated through the modulation of upstream PI3K, and its degree of phosphorylation indirectly reflects the levels of active PI3K. Phosphorylated PI3K recruits AKT and 3-phosphoinositide-dependent protein kinase 1 (PDK1) to the cell membrane and activates AKT. The phosphorylation of AKT directly stimulates mTOR, which is a ubiquitous serine-threonine protein kinase. Subsequently, the mTOR signaling pathway is activated in the brain parenchyma^23^,^27^,^29^, where it modulates pro- or anti-inflammatory cytokine synthesis in immune cells^24^ such as neutrophils^30^, macrophages^25^ and microglia^23^. The above activated pathway results in increased production of pro-inflammatory cytokines (e.g., TNF-α and IL-1β) to recruit neutrophils and monocytes^25^,^30^ and to activate microglia^23^. Inhibition of the mTOR pathway is uniquely poised to curtail the production of pro-inflammatory cytokines^23^,^25^,^30^,^31^ and to improve neurobehavioral deficits^27^,^29^,^31^.

As a result of empirical clinical practice over many centuries and the fact that traditional Chinese medicine (TCM)-derived compounds have numerous targets rather than using the one compound/one-target drug discovery paradigm^32^, accumulating research suggests that TCM is a potentially powerful novel drug. Xuefu Zhuyu decoction (XFZY) is an ancient TCM formula for the treatment of cardiac-cerebral vascular disease, including unstable angina pectoris^33^, ischemic heart disease^34^, hypoxic-ischemic brain injury^35^, and post-craniocerebral traumatic mental disorders^36^. It was first described in the book, “Yilin Gaicuo” by Qingren Wang in the late Qing Dynasty, approximately 185 years ago. At that time, it consisted of 11 crude herbs: *Prunus persica (L.) Batsch (Taoren), Angelicae sinensis (Oliv.) Diels (Danggui), Ligusticumi chuanxiong Hort. (Chuanxiong), Carthamus tinctorius L. (Honghua), Paeonia lactiflora Pall. (Chishao), Rehmannia glutinosa Libosch. (Dihuang), Citrus aurantium L. (Zhiqiao), Bupleurum chinense DC. (Chaihu), Platycodon grandiflorum (Jacq.) A. DC. (Jiegeng), Achyranthes bidentata Bl. (Niuxi)* and *Glycyrrhiza uralensis Fisch. (Gancao).* Randomized controlled trials of XFZY have demonstrated valid effects in patients with TBI^36^,^37^,^38^. XFZY*-*derived compounds, including *Hydroxysafflor yellow* A (HSYA) and Amygdalin, have been shown to possess an ability to control the inflammatory response^39^,^40^,^41^. The above evidence also indicates that the potential mechanism of neuroprotection against TBI by XFZY could involve the reduction of the inflammatory cascade. Nevertheless, details concerning the pharmacological anti-inflammatory activity of XFZY in TBI remain unknown.

The goal of the present study was to investigate whether XFZY exerts anti-inflammatory effects against TBI by inhibiting the PI3K-AKT-mTOR signaling pathway and to provide evidence for a potential role of XFZY as a neuroprotective drug for the treatment of TBI (experimental details are shown in a flow diagram in Fig. 1).

## Results

### Molecular characterization of XFZY using liquid chromatography coupled to a high-resolution ion trap and time-of-flight mass spectrometry (LCMS-IT-TOF)

To ensure the quality of the XFZY (Fig. 2A) that was used in our study, HSYA and Amygdalin originating from the monarch drug in prescription of XFZY were used as the central compounds. They were used as quality control standards for XFZY. LCMS-IT-TOF was performed with 0.1% formic acid in water and acetonitrile for separation. Coupled tandem mass spectrometry was used for the quantitative and qualitative analyses. As shown in Fig. 2, MS^1^ and MS^2^ of Amygdalin were *m/z* 458.16 (Fig. 2C) and 296.11 (Fig. 2D), respectively. MS^1^ and MS^2^ of HSYA were *m/z* 613.17 (Fig. 2E) and 451.12 (Fig. 2F), respectively. The retention times of Amygdalin and HSYA in the LCMS-IT-TOF chromatograms of XFZY in the positive ESI mode were 2.50 ± 0.02 min and 3.48 ± 0.04 min, respectively (Fig. 2B). The overall intra- and inter-day variations in HSYA and Amygdalin were less than 5%. The method was verifiable and provided good precision. A recovery test was conducted as an accuracy test, and the recovery of the two analytes was >90%. The results showed that the contents of two tested compounds in XFZY were as follows: HSYA, 0.942 ± 0.021 mg/g; Amygdalin, 0.107 ± 0.005 mg/g.

### XFZY improved neurological recovery after TBI

To investigate whether treatment with XFZY could improve neural functional recovery after TBI, we examined the changes in mNSS and calculated the variation in ΔmNSS over time. Following CCI, neurologic deficits were tested at predetermined time points, and treatment with XFZY resulted in an improved neurological recovery, as demonstrated by a decrease in mNSS (Fig. 3A) and an increase in ΔmNSS (Fig. 3B) scores, compared with the vehicle group. Rats that were treated with 9 g/kg XFZY continued to display significant improvements from the 3^rd^ to the 21^st^ day compared with the vehicle group, whereas on the 3^rd^ and 21^st^ days did the rats that were treated with 18 g/kg XFZY show a significant improvement in neurological recovery compared with the vehicle group, as indicated by both mNSS and ΔmNSS scores. Moreover, the two XFZY groups differed significantly from one another over the following intervals: 1–7, 1–14, and 1–21 days (Fig. 3B). Interestingly, these results indicated that greater improvements in neurological function were achieved with 9 g/kg XFZY than with 18 g/kg XFZY treatment.

### XFZY enhanced spatial memory acquisition after TBI

The Morris water maze (MWM) test was performed using sham-injured and CCI rats that were treated with vehicle, 9 g/kg XFZY, or 18 g/kg XFZY. We observed a significant effect of CCI on hidden platform trails (p < 0.01 for CCI versus sham injury) using a mixed-model RM ANOVA to determine the injury status (sham versus CCI) and three treatment groups (9 g/kg XFZY, 18 g/kg XFZY, or vehicle) without interaction. In sham-injured rats, treatment with 9 g/kg XFZY or 18 g/kg XFZY resulted in no significant differences in performance in the hidden or visible platform test compared with vehicle treatment (Fig. 4A) or probe trials (Fig. 4B). This finding suggests that XFZY had no significant effect on cognitive function in the absence of impairment by TBI. After CCI, significant differences between treatment groups were observed (P < 0.01), and rats treated with 9 g/kg XFZY exhibited significantly improved performance in the hidden platform (P < 0.05 versus vehicle) (Fig. 4C) and probe trials (P < 0.05 versus vehicle) (Fig. 4D). However, a significant change between the 18 g/kg XFZY and vehicle group was observed only in the probe trial (Fig. 4D), without significant differences in the hidden platform trial (P > 0.05 versus vehicle) (Fig. 4C). This finding indicated that treatment with 9 g/kg XFZY was more beneficial for the treatment of cognitive impairment induced by TBI than18 g/kg XFZY.

### XFZY significantly reduced the TBI-induced increase in arachidonic acid (AA) levels in the brain

Next, we assessed AA levels in injured brain tissues, both quantitatively and qualitatively, using gas chromatography-mass spectrometry (GC-MS). The *m/z* of AA was 67, 73, 75, 79, 91 and 117 (Fig. 5A). The retention time in GC-MS TICs of AA after MeOx-TMS derivatization was 24.90 ± 0.02 min (Fig. 5B). The contents of AA in the brain samples were quantitatively determined based on the ratio of the peak area to the authentic reference AA. Despite the gradual decrease in AA levels in TBI tissue, the levels of AA in ipsilateral brain tissue increased significantly from day 1 to 14. Treatment with 9 g/kg XFZY ameliorated the increase in AA after TBI throughout the entire study, whereas 18 g/kg XFZY was effective for only a short time (Fig. 5C). Moreover, on the third day, a significant reduction in AA was observed in ipsilateral brain tissue in rats that received 9 g/kg XFZY compared to 18 g/kg XFZY (Fig. 5C).

### XFZY markedly restrained the TBI-induced generation of pro-inflammatory factors in the brain

To target the pro-inflammatory factors TNF-α and IL-1β, which reflect the inflammatory status of the brain, we examined the levels of TNF-α and IL-1β in ipsilateral cerebral tissue following traumatic injury in the rodent model of CCI. The substantially upregulated TNF-α and IL-1β levels persisted in the ipsilateral cerebral tissue from the 1^st^ to the 14^th^ day post-TBI (Fig. 6). The maximal level of TNF-α was observed on the 3^rd^ day following TBI (Fig. 6A), and the peak values of IL-1β were detected on the 1^st^ day (Fig. 6B). Administration of XFZY (9 g/kg and 18 g/kg) significantly inhibited the increase in both TNF-α and IL-1β caused by TBI (Fig. 6), thereby implying that XFZY modulated the production of pro-inflammatory cytokines. Surprisingly, the extensive amelioration of the up-regulated TNF-α (Fig. 6A) levels and marked inhibitory effects of 9 g/kg XFZY on TNF-α (Fig. 6A) or IL-1β (Fig. 6B) were greater than those of 18 g/kg XFZY, which indicated that 9 g/kg XFZY more effectively downregulated the pro-inflammatory response.

### XFZY inhibited the phosphorylation activation of AKT, mTOR and p70S6K in the brain after TBI

To explore whether the anti-inflammatory effect of XFZY was regulated via the PI3K/AKT/mTOR signaling pathway, we performed a Western blot analysis of phosphor- or total AKT/mTOR/p70S6K protein expression in the ipsilateral hemisphere of sham-injured, CCI, or CCI-treated-with-XFZY rats. We observed a marked upregulation of AKT phosphorylation in ipsilateral brain tissue on the 1^st^ and 3^rd^ days after TBI (Fig. 7B), with no notable changes in total AKT expression (Fig. 7B). Analogously, increased phosphorylation of mTOR was observed from the 1^st^ to the 14^th^ day (Fig. 7D) and was accompanied by an absence of remarkable variation in the protein expression of total mTOR (Fig. 7D). The p70 ribosomal S6 kinase (p70S6K), one of the main downstream targets of mTOR signaling, was also significantly phosphorylated in the ipsilateral hemisphere from the 1^st^ to the 14^th^ day after TBI (Fig. 7F). There was no significant change in total p70S6K expression. The increased phosphorylation of AKT/mTOR/p70S6K suggested that the PI3K/AKT/mTOR signaling pathway was activated in the post-TBI brain.

Treatment with 9 g/kg XFZY dramatically diminished the increased phosphorylation of AKT/mTOR/p70S6K in the ipsilateral hemisphere of TBI rats throughout the experiment (Fig. 7B,D,F), whereas 18 g/kg XFZY occasionally exerted a similar inhibition of AKT/mTOR/p70S6K phosphorylation (Fig. 7B: p-AKT, on the 1^st^, 3^rd^ and 14^th^ day; Fig. 7D: p-mTOR, on the 1^st^ and 3^rd^ day; Fig. 7F: p-p70S6K, on the 1^st^ day). We also found that 9 g/kg XFZY markedly reduced the phosphorylation of AKT/mTOR/p70S6K at certain time points (Fig. 7B: p-AKT, on the 7^th^ and 14^th^ day; Fig. 7D: p-mTOR, on the 1^st^, 7^th^ and 14^th^ day; Fig. 7F: p-p70S6K, on the 3^rd^, 7^th^ and 14^th^ day). The above findings demonstrate that the inhibition of the PI3K-AKT-mTOR signaling pathway was more pronounced following treatment with 9 g/kg XFZY than 18 g/kg XFZY. In addition, there were almost no remarkable differences in total AKT/mTOR/p70S6K expression after treatment with 9 g/kg or 18 g/kg XFZY compared with the Vehicle group, except for the decreased levels of total AKT/p70S6K protein in response to 9 g/kg XFZY on the 1^st^ day and the increased total AKT expression in response to 18 g/kg on the 14^th^ day (Fig. 7B,F).

## Discussion

The inflammatory response is one of the leading causes of secondary damage during both the acute and chronic phases of TBI, especially in brain contusions^12^,^18^. In this study, it was primarily found that the administration of XFZY after TBI facilitated the recovery of neurological deficits and improved cognitive function. Furthermore, a reversal of the increase in AA, pro-inflammatory factor (TNF-α and IL-1β) and phospho-AKT/mTOR/P70S6K expression in the ipsilateral brain tissue was observed in TBI rats. These results showed that XFZY provided significant neuroprotection against TBI via anti-inflammatory effects resulting from the inhibition of the activated PI3K-AKT-mTOR signaling pathway. Intriguingly, the classical dose of XFZY (i.e., 9 g/kg in rats), as defined by Qingren Wang from “Yilin Gaicuo” provided better curative effects than the ultra-routine dose (i.e., 18 g/kg in rats), which indicated that the classical dose used in TCM is scientific and reasonable.

Inflammation, which is well known as a double-edged sword, consists of initial, perpetual and resolution phases. Adaptive inflammation is helpful for tissue repair, whereas excessive levels of inflammation result directly in secondary damage^19^. Arachidonic acid (20:4ω6), a premier eicosanoid precursor, is maintained at a steady cellular level by a host of enzymes. In the TBI model, in response to mechanical trauma, cells are activated to release excessive AA from membrane lipids, which are mobilized by phospholipases (PLA_2_)^42^. Subsequently, in mammalian cells, AA is metabolized into prostaglandins and leukotrienes by cyclooxygenase-2 (COX-2) and 5-lipoxygenase (5-LO), respectively^19^,^42^. Novel synthetic prostaglandins, such as prostaglandin E2 (PGE2) and prostacyclin (PGI2), contribute to inflammatory infiltration, including the leakage of polymorphonuclear leukocytes (i.e., neutrophils) and monocytes^42^. The generated leukotriene B4 (LTB4) is transferred out of the cell membrane as an inflammatory chemotactic agent to stimulate inflammatory cell aggregation, which exerts damaging effects in certain locations^43^. The above processes are exacerbated by the disruption of the blood-brain barrier in TBI. Moreover, the abundant prostaglandins and leukotrienes that originate from AA result directly in chronic inflammation^19^. In the present study, the increase in AA in ipsilateral brain tissue persisted from the 1^st^ to the 14^th^ day after TBI and decreased gradually over time (Fig. 5C), as supported by the increased serum levels of AA observed in the TBI metabonomic research reported by Shuguang Yang *et al*^44^. These results suggest that inflammation persisted continuously until at least the 14^th^ day post-TBI. Fortunately, treatment with XFZY significantly reversed the increased AA levels, and a dosage of 9 g/kg was more effective than 18 g/kg.

During TBI, peripherally derived inflammatory cells can provide a neuroprotective effect or aggravate maladaptive secondary injury reactions over time. First, recruited neutrophils that are localized to the area of brain damage are activated to release metalloproteinases, proteases, TNF-α, and ROS^12^, further accelerating the recruitment of monocytes to the damaged area^20^, as well as the activation of macrophages and microglia^45^. After migrating to the area of the brain lesion, monocytes, multipotent bone marrow-derived leukocytes become macrophages, which are related to the efferocytosis of apoptotic granulocytes^19^. Following TBI, microglia, resident innate tissue macrophages in the CNS, are converted into the M1 phenotype. This phenotype possesses an analogous appearance and functionality to monocyte-derived macrophages^46^, which are characterized by a pro-inflammatory response and antimicrobial activities^47^. In addition, pernicious substances that are released by neuronal or glial death exacerbate the secondary injury, thereby establishing a vicious cycle of events. These pathophysiologic processes give rise to a host of cytokines, ROS, purines, growth factors, excitatory amino acids and damage-associated molecular pattern molecules (DAMPs)^12^, which can activate a diverse group of signaling pathways under the hyperactivated state induced by TBI.

Emerging evidence suggests that the PI3K-AKT-mTOR signaling pathway is activated in the brain parenchyma following TBI^27^,^28^,^29^. AKT is mainly activated through the modulation of upstream PI3K, and its degree of phosphorylation indirectly reflects the levels of active PI3K. Following TBI, stimulation of BCR, TCR, cytokine receptors and toll-like receptors (TLRs) by signals that are released in response to brain trauma activate tyrosine kinase adaptor molecules that are present on the cell membrane to recruit the p85 subunit of PI3K, which contributes to the activation of PI3K to catalyze the transformation of phosphatidylinositol 4,5-bisphosphate (PIP2) to phosphatidylinositol 3,4,5-triphosphate (PIP3)^24^. PIP3, as a second messenger, recruits AKT and PDK1 to the cell membrane and phosphorylates downstream targets, including AKT. Consequently, AKT is activated by PDK1. The phosphorylated AKT results in the direct activation of mTOR by the proline-rich AKT/PKB substrate 40 kD (PRAS40) or the indirect activation by tuberous sclerosis protein complex (TSC) and Ras homolog enriched in brain (Rheb)^8^,^48^. mTOR, a ubiquitous serine-threonine protein kinase, regulates various important cellular functions that are involved in transcription, mRNA turnover, translation, and protein stability. The regulation of translation is its best-characterized function in mammalian cells^49^. The p70 ribosomal S6 kinase (p70S6K) is a key substrate for controlling the ratio of mRNA translation^50^. In our study, although a remarkable increase in AKT phosphorylation was observed on the 1^st^ and 3^rd^ days after TBI, phosphorylation of mTOR/p70S6K, the downstream targets of AKT, was significantly increased in CCI-treated rats from the 1^st^ to the 14^th^ day (Fig. 7), which suggested that the PI3K-AKT-mTOR signaling pathway was activated in the injured ipsilateral hemisphere from the 1^st^ to the 14^th^ day. An increasing number of studies have demonstrated that the activated PI3K-AKT-mTOR signaling pathway results in increased production of pro-inflammatory cytokines (e.g., TNF-α and IL-1β) and the recruitment of neutrophils and monocytes^25^,^30^, as well as activated microglia^23^.

It is evident that TBI induces amplified and prolonged neuroinflammation that negatively influences cognitive and behavioral processes. TNF-α as well as rises in IL-1β have been described in TBI, and associated adverse events and, potentially, can lead to secondary neuronal damage^51^,^52^. The both are the key cytokines as master regulators of neuroinflammatory processes post TBI^51^,^52^. Over-production of TNF-α and IL-1β in chronic neuroinflammation following TBI may contribute to disruption of synaptic and plasticity deficits of cognitive function^1^. So reduction of elevated TNF-α and IL-1β can restore neuronal function and reverse cognitive deficits post TBI. In the present study, TNF-α and IL-1β were significantly upregulated in ipsilateral brain tissue from the 1^st^ to the 14^th^ day after TBI (Fig. 6). Furthermore, we observed maximal levels of TNF-α and IL-1β on the 3^rd^ and 1^st^ days, respectively, following TBI. These results are consistent with previous reports^10^,^22^. The increased levels of pro-inflammatory cytokines (e.g., TNF-α and IL-1β) that were released in the brain after TBI resulted in the expansion of secondary damage. Inhibition of the up-regulation of the PI3K-AKT-mTOR signaling pathway dramatically suppressed the production of pro-inflammatory cytokines (e.g., TNF-α and IL-1β) in brain tissues^23^,^31^, and this effect was accompanied by improvements in the post-injury behavioral deficits, including neurological and cognitive functions^23^,^28^,^49^. This phenomenon may be related to one of the molecular mechanisms that may be attributable to the over-production of TNF-α and IL-1β in neuroinflammation during TBI. Superfluous TNF-α during neuroinflammation contributes to deficits in cognitive function through the reduction of postsynaptic NMDAR currents^53^, which is further supported by the ability of TNF-α synthesis inhibition to restore neuronal function and reverse cognitive deficits induced by neuroinflammation^53^,^54^. Analogously, a sustained elevation of IL-1β levels in the hippocampus induced the impairment of spatial memory^55^, while neutralization of IL-1β in the CCI model improved cognitive outcomes^56^. Similar results were observed in our study following treatment with XFZY (9 g/kg and 18 g/kg), which inhibited the activated PI3K-AKT-mTOR signal pathway by reducing the phosphorylation levels of AKT/mTOR/p70S6K (Fig. 7), thereby diminishing the over-production of TNF-α and IL-1β (Fig. 6) and alleviating the impairment of neurological outcomes (Fig. 3) and of spatial memory (Fig. 4). The above results support the speculation that XFZY significantly improved behaviors (neurological or cognitive functions) through suppressing the over-production of pro-inflammatory cytokines by inhibiting the activated PI3K-AKT-mTOR signaling pathway.

This is the first study to elucidate the neuroprotective mechanism underlying the anti-inflammatory effects of XFZY in a rat model of TBI (Fig. 8). XFZY reversed the increased AA levels caused by trauma, implying that the recruitment of inflammatory cells by prostaglandins or leukotrienes was reduced in the brain injury lesion. Inhibition of the PI3K-AKT-mTOR signaling pathway by XFZY resulted in reduced production of pro-inflammatory cytokines, potentially due to effects on activated neutrophils, monocytes, microglia and/or other factors in the injured brain parenchyma after TBI. The effect of XFZY on the neutralization of the imbalance of pro-inflammatory cytokines was beneficial for improvements in neurological and cognitive functions.

Notably, the neuroprotection conferred by the classical dose of XFZY (9 g/kg) was superior to an ultra-routine dose (18 g/kg) in its ability to both reduce inflammatory mediators and improve behavioral performance, indicating that the classical dose of the TCM prescription, which is based on long-term clinical practice, is scientific and reasonable. In the present study, 9 g/kg was defined as the classical dose of XFZY according to the principle that the dose in the rat is 6.7 times than that in the human, as described in “Yilin Gaicuo”^57^. We doubled the classical dose (18 g/kg) as ultra-routine dose to compare the different curative effects. These are the first results to demonstrate that the classical dose of XFZY had more beneficial effects than the ultra-routine dose in terms of neuroprotection against TBI. These results partially elucidate an optimal dose of XFZY for neuroprotective effects, considering that the XFZY complex consists of thousands of compounds and that changes in the dose may influence the interactions of absorbed active compounds *in vivo* according to the multi-target therapy^32^. The classical dose may be in close proximity to the optimal dose compared with the ultra-routine dose, but details regarding the corresponding mechanism require further confirmation in an ongoing study.

This study has several limitations. Although it is universally acknowledged that XFZY is a classical TCM, which may have dozens of different chemical compositions, the absorbed bioactive compositions derived from XFZY that exerted protective functions in the brain remain unclear. Furthermore, the manner by which the activated PI3K-AKT-mTOR signaling pathway was inhibited in inflammatory cells in the cerebral parenchyma following TBI is unknown. Additional research is required to elucidate the mechanisms underlying these phenomena.

In summary, XFZY exerts neuroprotective effects by neutralizing the neuroinflammation caused by TBI and ameliorating the effects on neurological and cognitive functions post-injury by inhibiting the PI3K-AKT-mTOR signaling pathway. This is the first study to verify the superiority of the classical dose of XFZY compared with an ultra-routine dose for the treatment of TBI from the perspective of anti-inflammatory pathways, which are more valuable for the clinical application of XFZY for the treatment of patients with TBI. The present study demonstrated that XFZY could be a potentially promising therapeutic option for the treatment of TBI.

## Methods

### XFZY decoction preparation

The extracts of XFZY decoction (*Prunus persica (L.) Batsch, Carthamus tinctorius L., Angelicae sinensis (Oliv.) Diels, Rehmannia glutinosa Libosch., Achyranthes bidentata Bl., Paeonia lactiflora Pall., Citrus aurantium L., Glycyrrhiza uralensis Fisch., Ligusticumi chuanxiong Hort., Platycodon grandiflorum (Jacq.) A. DC., and Bupleurum chinense DC.* at a ratio of 8:6:6:6:6:4:4:4:3:3:2) were prepared for crude plant medicines. They were purchased from the Pharmacy of Xiangya Hospital of Central South University, Hunan Province, PR China. Each herb was authenticated by the herbal medicinal botanist, Professor SY Hu, at the Department of Chinese herbal medicine of Central South University. We kept the voucher specimens (NO. 20140039) at Xiangya Hospital of Central South University (Changsha, China). The eleven herbs of XFZY with the above ratios were used to generate a lyophilized powder of XFZY according to a standard process^58^. Finally, 1 g of the lyophilized powder was determined to contain 5.2 g of crude herbs. It was stored at 4 °C before being resolved with distilled water for usage, according to the standard of 1 g/ml (*w/v*).

### HSYA and amygdalin determination in XFZY using LCMS-IT-TOF

The Shimadzu LCMS-IT-TOF system (Tokyo, Japan) was used in positive ion mode with an electrospray ionization (ESI) source. A Shim-Pack XR-ODS (C18 column (2.0 mm i.d. ×75 mm, 1.6 μm, Shim) was employed for chromatography separation, and 0.1% formic acid in water (A) and acetonitrile (B) at a flow rate of 0.4 mL/min was used to establish the gradient elution (17% B for 0–2.0 min, 17%–35% B for 2.0—3.0 min, 35%–44% B for 3.0–7.0 min, 44%–55% B for 7.0–9.0 min, 55%–60% B for 9.0–14.0 min, 60%–63% B for 14.0–17.0 min, and 63%-68% B for 17.0–19.0 min). The column temperature was set at 40 °C. The sample injection volume was 5 μL. The detection wavelengths ranged from 190 nm to 800 nm. The following parameters were used for analytical MS: interface voltage, 4.5 kV; ESI voltage, 1.6 kV; nebulizing gas (N_2_) flow rate, 1.5 L/min; ion trap pressure, 1.8 × 10^−2^ Pa; interface temperature, 200 °C; drying gas pressure, 100 kPa; ion accumulation time, 30 ms. Ultra-high purity argon was applied for collision-induced dissociation (CID). The above-mentioned standard solution of 1 g/ml XFZY was centrifuged for 10 min (16,000 rpm, 4 °C). The upper layer was filtered through a 0.22-μm nylon filter. The leachate was diluted twice with distilled water and then injected into the LCMS-IT-TOF.

### Animals

Male Sprague-Dawley (SD) rats weighing 200–250 g were supplied by the Laboratory Animal Research Center of Central South University. They were maintained under standard conditions for at least 1 week and then fasted for 12 h with free access to food and water before each experiment. All of the animal experiments were approved by the Central South University Animal Ethics Committee and conformed to the Guidelines for the Care and Use of Laboratory Animals.

### Controlled Cortical Impact Model of TBI

The rat model of Controlled Cortical Impact (CCI) was performed using an electronic controlled pneumatic impact device (TBI 0310, Precision Systems & Instrumentation, Fairfax Station, VA) according to a previous report^58^. The parameters sued for this apparatus were as follows: depth of impact, 5.0 mm from the cortical surface; impact velocity, 6.0 m/sec; dwell time, 500 msec. The rats were intraperitoneally anesthetized with 3% pentobarbital (50 mg/kg). Under sterile conditions, a midline longitudinal incision was created over the skull, and a 5-mm craniotomy was generated using a portable drill and trephine over the left parietal cortex (the center of the coordinates of the craniotomy relative to the bregma: 1 mm posterior, 1 mm lateral), and the bone flap was removed. The rats were then subjected to CCI in the TBI group, whereas no cerebral cortex trauma was applied in the sham group. The scalp was closed using cyanoacrylate tissue glue. Throughout the surgery, the body temperature of the rats was monitored and maintained at 37.0 ± 0.5 °C.

### Experimental design and sample collection

**Figure 1:**
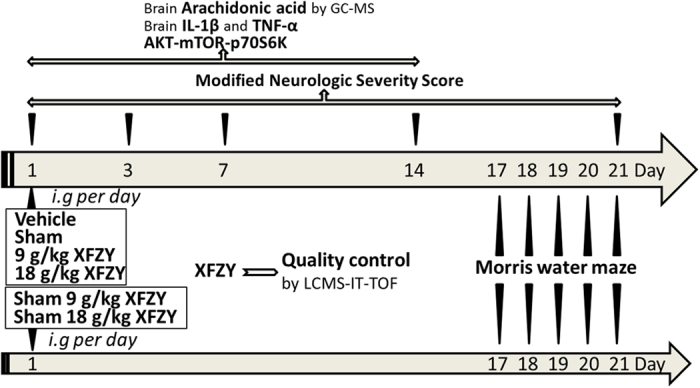
Flow diagram of the experiment. The experiment was performed on six groups: Vehicle, Sham, 9 g/kg XFZY, 18 g/kg XFZY, Sham 9 g/kg XFZY and Sham 18 g/kg XFZY. The dosage regimen for each group was applied continuously once per day after TBI. The quality control of XFZY was performed using LCMS-IT-TOF. On the 1^st^, 3^rd^, 7^th^ and 14^th^ day post-TBI, 8 rats were randomly drawn from each group, except for the sham 9 g/kg XFZY and sham 18 g/kg XFZY groups. The rodents were then sacrificed for sample collection after the neurological function tests. Brain samples were used for biochemical assays and determination of the arachidonic acid by GC-MS. The remaining 12 rats in each above-mentioned group were examined to evaluate the neurological function scores on the 1^st^, 3^rd^, 7^th^, 14^th^ and 21^st^ days after TBI. These 12 rats in each group and 8 rats in each of the sham XFZY groups (9 g/kg or 18 g/kg) underwent MWM testing from the 17th to the 21st day.

**Figure 2:**
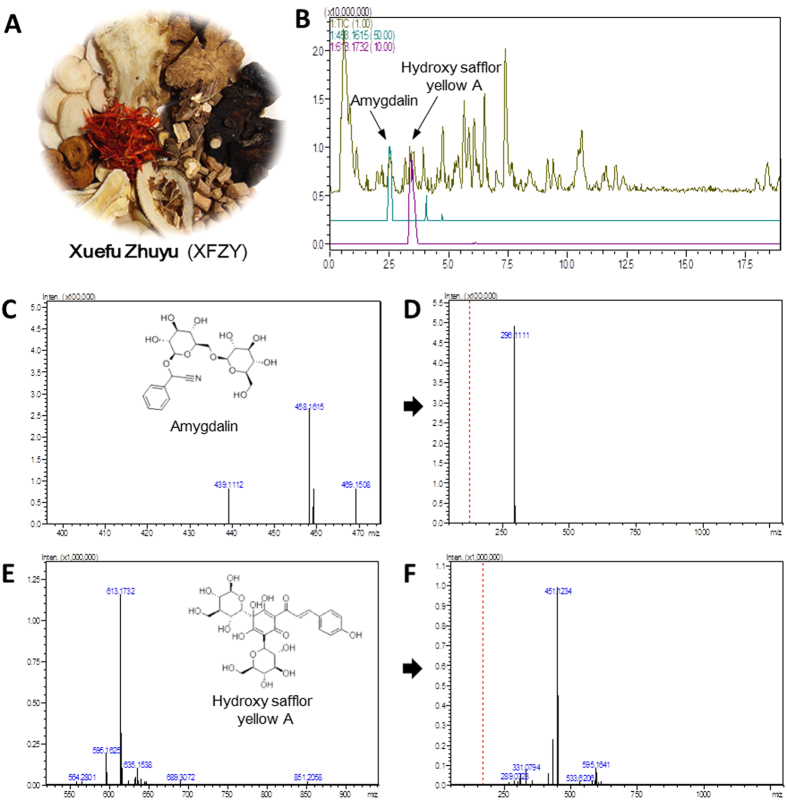
Liquid chromatography coupled to high-resolution ion trap and time-of-flight mass spectrometry (LCMS-IT-TOF) analysis for HSYA and Amygdalin determination, which originated from the monarch drug of XFZY. (**A**) Prepared Chinese herbal medicines of XFZY in small amounts that are ready for decoction. (**B**) LCMS-IT-TOF TIC chromatograms of XFZY in positive ESI mode and chromatographic profiles of Amygdalin and HSYA. (**C**,**D**) MS^1^ and MS^2^ of Amygdalin were *m/z* 458.16 and 296.11, respectively. (**E, F**) MS^1^ and MS^2^ of HSYA were *m/z* 613.17 and 451.12, respectively.

**Figure 3:**
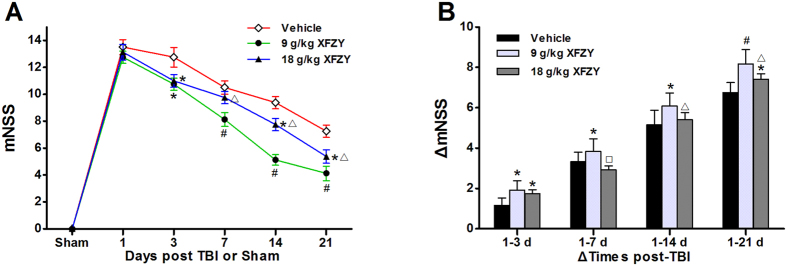
Modified neurologic severity score (mNSS) after TBI or sham injury or TBI with XFZY. (**A**) Neurological function was evaluated by mNSS after 1 hour to assess the initial disability and on the 3^rd^, 7^th^, 14^th^, and 21^st^ days after TBI. Treatment with 9 g/kg XFZY significantly lowered the mNSS on the 3^rd^, 7^th^, 14^th^, and 21^st^ days compared with the Vehicle group. Treatment with 18 g/kg XFZY resulted in a significant decrease on the 3^rd^ and 21^st^ days (n = 8/group, data are analyzed by two-way ANOVA and presented as the mean ± SEM. p < 0.05 vs. the Vehicle group). (**B**) Changes in mNSS (ΔmNSS) were assessed at various time intervals between the 1^st^ day and multiple predetermined time points thereafter, considering that the performance of mNSS was not significant on the 1^st^ day (mean mNSS values: 9 g/kg XFZY group = 12.8 ± 0.4, 18 g/kg XFZY group = 13.2 ± 0.4, Vehicle group = 13.5 ± 0.5, all p > 0.05 compared with each other). Treatment with 9 g/kg XFZY significantly enhanced ΔmNSS over time intervals of 1–3, 1–7, 1–14 and 1–21 days. Treatment with 18 g/kg XFZY significantly increased ΔmNSS during the interval of 1–3 and 1–21 days. The ΔmNSS in the 9 g/kg XFZY group increased significantly during the time intervals of 1–7, 1–14, and 1–21 days compared with the 18 g/kg XFZY group (n = 12/group, data were analyzed with RM ANOVA and are presented as the mean ± SEM. *p < 0.05, ^#^p < 0.01 vs. the Vehicle group. ^∆^p < 0.05, ^□^p < 0.01 vs. 9 g/kg XFZY group).

**Figure 4:**
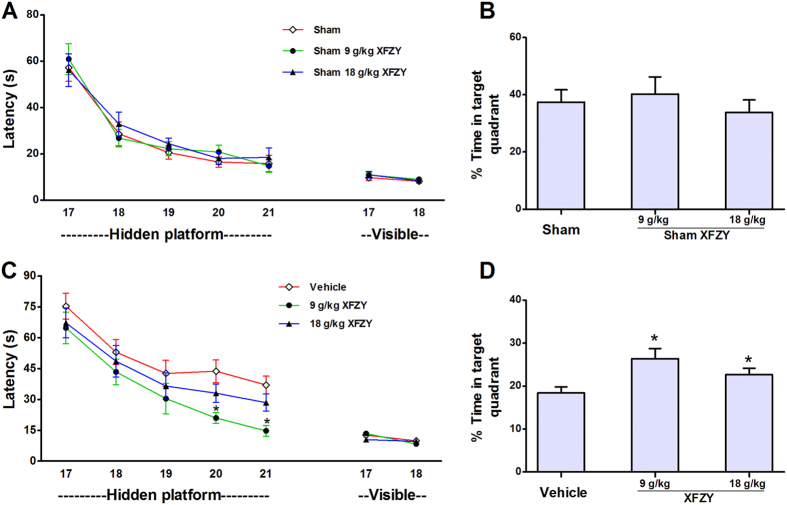
Effect of XFZY on cognitive outcomes after sham injury or controlled cortical impact (CCI). (**A**,**B**) Sham-injured rats (n = 8/group) were gavaged with 9 g/kg XFZY, 18 g/kg XFZY, or an equivalent amount of saline each day. The Morris water maze (MWM) test was then performed from the 17^th^ to the 21^st^ day after the sham injury. Performance in the MWM revealed no differences between the hidden and visible platform (p > 0.05 for group, repeated-measures ANOVA (RM ANOVA)) or in the probe trials (p > 0.05, **B**), despite learning the task (p < 0.01 for time, hidden and visible platform trials). (**C, D**) In the CCI groups (n = 12/group), significant group effects were observed in the hidden platform tests to assess the impact of XFZY treatment (p < 0.01 for the group) with learning the hidden platform paradigms (p < 0.01 for time in each group). The rats that were treated with 9 g/kg XFZY performed significantly better than the vehicle-treated mice in the hidden platform (p < 0.05) and probe trials (p < 0.05). Although there were no significant differences in the hidden platform test, the 18 g/kg XFZY group displayed significantly improved performance in the probe trials compared with the Vehicle group (p < 0.05). No differences in visible platform performance were noted among the CCI groups (p > 0.05 for group). *p < 0.05 versus the Vehicle group.

**Figure 5:**
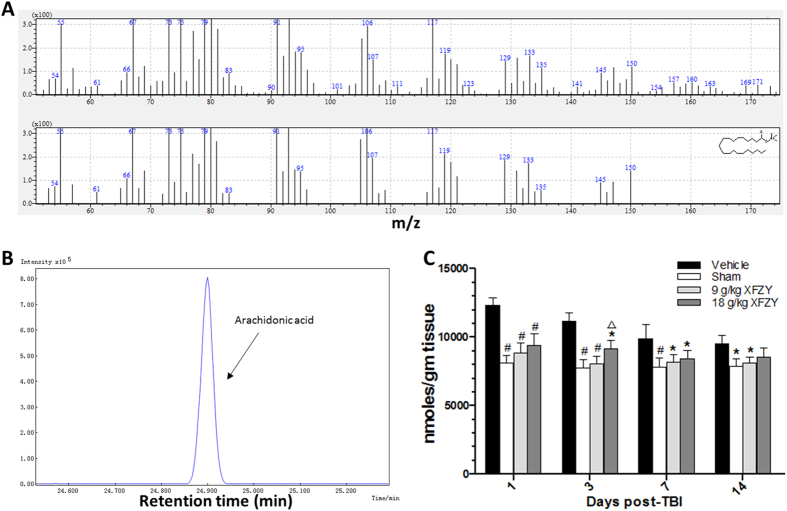
Quantitative determination of Arachidonic acid (AA) in ipsilateral brain tissues on the 1^st^, 3^rd^, 7^th^, 14^th^ and 21^st^ days after TBI or sham injury or TBI gavaged with oral XFZY. (**A**) GC-MS *m/z* of AA after MeOx-TMS derivatization is 67, 73, 75, 79, 91 and 117. The top diagram shows the mass spectrum of AA in a brain sample, and the bottom diagram shows the mass spectrum of standard AA. (**B**) GC-MS TICs of AA after MeOx-TMS derivatization. (**C**) Brain tissue concentration of AA on the 1^st^, 3^rd^, 7^th^, 14^th^ and 21^st^ days after injury. The contents of AA were significantly increased in the Vehicle group compared with the sham group from the 1^st^ to the 14^th^ days post-injury, whereas treatment with 9 g/kg XFZY significantly reduced the increased AA levels compared with the Vehicle group on the 1^st^, 3^rd^, 7^th^, and 14^th^ days. Treatment with 18 g/kg XFZY significantly reduced the increased levels of AA compared with the Vehicle group on the 1^st^, 3^rd^, and 7^th^ days. On the 3^rd^ day, treatment with 9 g/kg XFZY significantly reduced the levels of AA in ipsilateral brain tissue compared with 18 g/kg XFZY group (n = 8/group). All of the data were analyzed by two-way ANOVA and are presented as the mean ± SEM. *p < 0.05, ^#^p < 0.01 vs. the Vehicle group. ^Δ^p < 0.05 vs. the 9 g/kg XFZY group.

**Figure 6:**
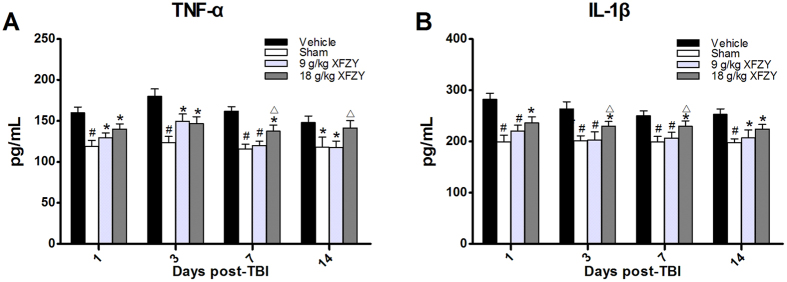
Assay of pro-inflammatory cytokine levels in brain tissue. (**A**) The levels of TNF-α in brain lysates from each group. The TNF-α levels were significantly increased in the Vehicle group compared with the Sham group from the 1^st^ to the 14^th^ day post-injury, whereas treatment with 9 g/kg XFZY significantly reduced the increase in TNF-α compared with the Vehicle group on the 1^st^, 3^rd^, 7^th^ and 14^th^ days. Treatment with 18 g/kg XFZY significantly reduced the increase in TNF-α levels compared with the Vehicle group on the 1^st^, 3^rd^ and 7^th^ days (n = 8/group). On the 7^th^ and 14^th^ days, the levels of TNF-α in the 9 g/kg XFZY group were significantly lower than those in the 18 g/kg XFZY group. (**B**) The levels of IL-1β in brain lysates in each group. The contents of IL-1β were significantly increased in the Vehicle group compared with the Sham group from the 1^st^ to the 14^th^ day post-injury. Treatment with 9 g/kg XFZY significantly reduced the increased levels of IL-1β compared with the Vehicle group on the 1^st^, 3^rd^, 7^th^, and 14^th^ days. Treatment with 18 g/kg XFZY significantly reduced the increased levels of IL-1β compared with the Vehicle group on the 1^st^, 3^rd^, 7^th^, and 14^th^ days. On the 3^rd^ and 7^th^ days, treatment with 9 g/kg XFZY significantly reduced the levels of IL-1β in the ipsilateral brain tissue compared with treatment with 18 g/kg XFZY (n = 8/group). All of the data were analyzed by two-way ANOVA and are presented as the mean ± SEM. *p < 0.05, ^#^p < 0.01 vs. the Vehicle group. ^∆^p < 0.05 vs. the 9 g/kg XFZY group.

**Figure 7:**
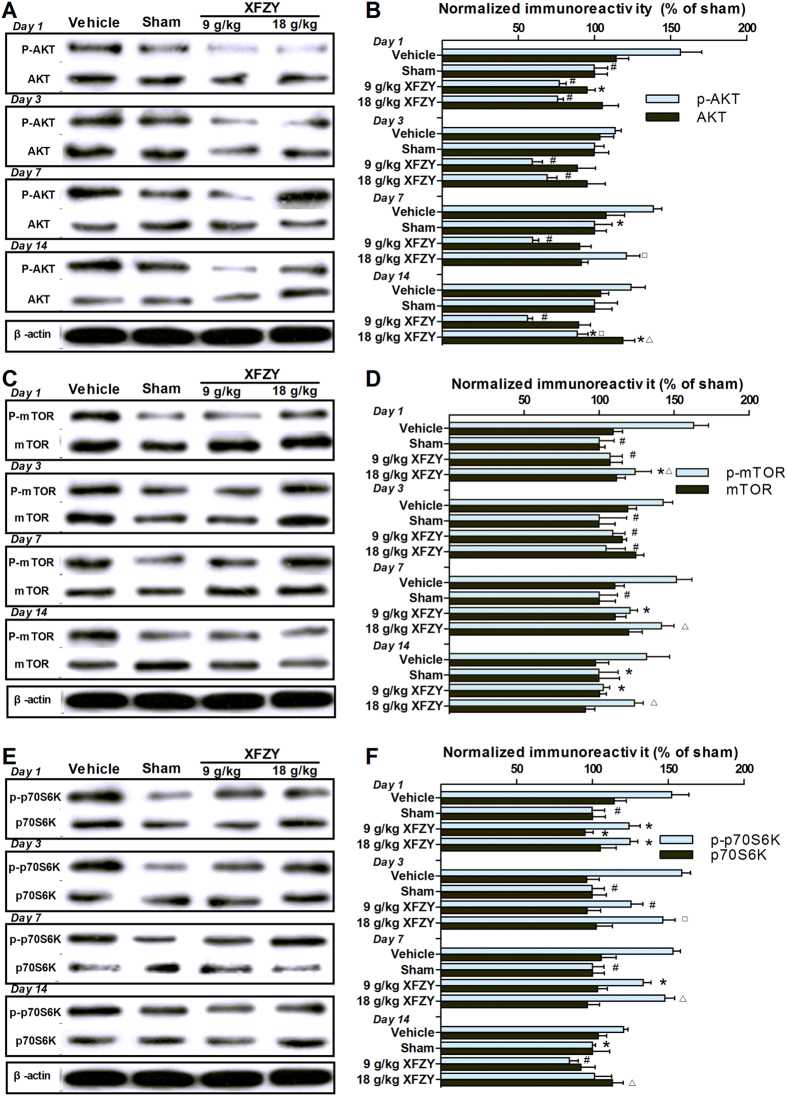
The effects of XFZY on the PI3K-AKT-mTOR signaling pathway after TBI. (**A**,**B**) The levels of p-AKT decreased significantly on the 1^st^ and 3^rd^ days in ipsilateral brain tissue after TBI. Treatment with 9 g/kg XFZY significantly reduced the increase in p-AKT expression from the 1^st^ to the 14^th^ day compared with the Vehicle group. The same trend was observed in the 18 g/kg XFZY group on the 1^st^, 3^rd^ and 14^th^ days. The expression levels of p-AKT were significantly lower in the 9 g/kg XFZY group compared with the 18 g/kg XFZY group on the 7^th^ and 14^th^ days. No significant changes were detected in total AKT expression between the Sham and Vehicle group. (**C**,**D**) p-mTOR was significantly increased from the 1^st^ to the 14^th^ day after TBI. Treatment with 9 g/kg XFZY significantly decreased p-mTOR expression compared with the Vehicle group from the 1^st^ to the 14^th^ day. Treatment with 18 g/kg XFZY significantly reduced p-mTOR on the 1^st^ and 3^rd^ days. mTOR expression in the 9 g/kg XFZY group was significantly reduced compared with the 18 g/kg XFZY group on the 1^st^, 7^th^ and 14^th^ days. There were no significant changes in total mTOR in comparisons between each group. (**E**,**F**) p-p70S6K increased significantly from the 1^st^ to the 14^th^ day after TBI. Treatment with 9 g/kg XFZY significantly reduced p-p70S6K expression on the above days. The same tendency was observed in the 18 g/kg XFZY group on the 1^st^ day. p-p70S6K expression was significantly reduced in the 9 g/kg XFZY group compared with the 18 g/kg XFZY group on the 3^rd^, 7^th^ and 14^th^ days. No significant changes were detected in total p70S6K expression between the Sham and Vehicle group. Data are the mean ratio between targeting proteins and β-actin and are presented as the percentage of XFZY (or Vehicle)-treated brains over the control Sham-treated brains. The data represent the mean fold induction ± SEM, as analyzed by ANOVA. *p < 0.05, ^#^p < 0.01 vs. the Vehicle group. ^∆^p < 0.05, ^□^p < 0.01 vs. the 9 g/kg XFZY group.

**Figure 8:**
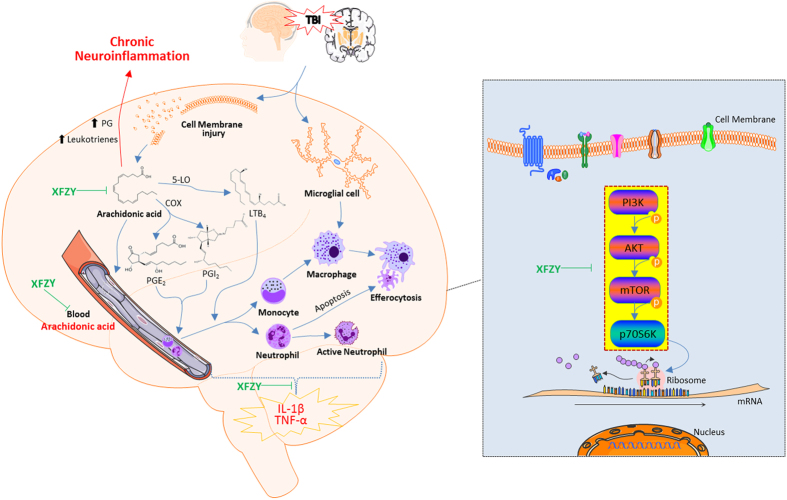
Inflammatory response in the brain after TBI and the pathomechanism of the anti-inflammatory effect of XFZY. Following TBI, the mechanical injury-stimulated cell membrane releases arachidonic acid (AA), which is metabolized into prostaglandin E2 (PGE2) and prostacyclin (PGI2) by cyclooxygenase-2 (COX-2) and into leukotrienes (LTB_4_) by 5-lipoxygenase (5-LO). The three inflammatory mediators initiate acute inflammation, including changes in blood flow, increased capillary permeability and inflammatory cell recruitment in the brain injury ambitus zone, including polymorphonuclear leukocytes (i.e., neutrophils) and monocytes. Excess prostaglandins and leukotrienes contribute to chronic inflammation. The neutrophils are activated to further release chemotactic factors, devour necrotic tissue and sterilize bacteria. The influx of monocytes and resident microglial cells develop into macrophages, which secrete pro-inflammatory factors (e.g., TNF-α and IL-1β) and consume foreign bodies, necrotic tissue or apoptotic cells (e.g., efferocytosis). XFZY significantly suppressed the increased levels of blood AA, TNF-α and IL-1β in brain tissue, indicating that XFZY possesses anti-inflammatory effects. To target the PI3K-AKT-mTOR signaling pathway, XFZY significantly reversed the elevated phosphorylation of AKT/mTOR in brain tissue post-TBI, as well as the downstream p70S6K, resulting in a reduced translation ratio of inflammatory factors and exerting anti-inflammatory effects.

As shown in Fig. 1, the rats were randomly sorted into six groups: (1) Sham-operated (sham, n = 44): rats that underwent the CCI procedure without cortex trauma and were gavaged with 0.9% NaCl; (2) TBI + Vehicle (Vehicle, n = 44): rats that underwent CCI and received the same amount of saline intragastrically; (3) TBI + 9 g/kg XFZY (9 g/kg XFZY, n = 44): CCI rats that received XFZY (9 g/kg) orally; (4) TBI + 18 g/kg XFZY (18 g/kg XFZY, n = 44): CCI rats that received XFZY (18 mg/kg) orally; (5) Sham + 9 g/kg XFZY (Sham 9 g/kg XFZY, n=8): rats from the sham group that received XFZY (9 g/kg) orally; (6) Sham + 18 g/kg XFZY (Sham 18 g/kg XFZY, n = 8): rats from the sham group that received XFZY (18 g/kg) orally. The dosage regimen for each group was applied continuously once per day after TBI. On the 1^st^, 3^rd^, 7^th^ and 14^th^ day post-TBI, 8 rats were randomly drawn from each group, except for the sham 9 g/kg XFZY and sham 18 g/kg XFZY groups. The rodents were then sacrificed for sample collection after the neurological function tests. The remaining 12 rats in each above-mentioned group were examined to evaluate the neurological function scores on the 1^st^, 3^rd^, 7^th^, 14^th^ and 21^st^ days after TBI. These 12 rats in each group and 8 rats in each of the sham XFZY groups (9 g/kg or 18 g/kg) underwent MWM testing from the 17^th^ to the 21^st^ day.

After execution by cervical dislocation, the ipsilateral hemispheres were rapidly removed. After rinsing away the surface blood with ice-cold 0.9% NaCl, the samples were quickly frozen in liquid nitrogen and stored at −80 °C until biochemical assays and determination of AA by GC-MS.

### AA determination in rat brain using GC/MS

Brain samples were extracted according to Hongfei Yue *et al*^59^, with modifications. Briefly, 20 mg of brain tissue pieces were mixed with 200 μL of methanol and 2 μL of formic acid. After homogenization using a homogenizer (TissueLyser LT, German), the mixtures were clarified by centrifugation. The upper layer was diluted in 1.8 mL distilled water. The supernatant was loaded onto the solid phase extraction (SPE) cartridges Oasis®HLB (Maliford, MA, USA), which were then washed with 3 mL distilled water and 1 mL 10% methanol and dried under vacuum conditions. The obtained analytes were evaporated to dryness with N_2_ gas. The dry extract was dissolved and vortexed, and the resultant mixture was constantly heated at 70 °C for 1 h. Next, 100 μL of N,O-bistrimethylsilyltrifluoroacetamide (BSTFA) and the catalyst 1% trimethylchlorosilane (TMCS) were added to the solution. Finally, the mixed solution was heated and diverted into the GC microvial for GC-MS analysis.

After extraction and derivatization, 1.0 μL of the sample solution was injected at a split ratio of 1:10 into a GC/MS (Kyoto, Japan). The column temperature was maintained at 70 °C for 4 min and then increased to 300 °C in increments of 1.0 mL/min and maintained for 3 min. The injection temperature was set at 280 °C, with unbolting of the septum purge at a flow rate of 3 mL/min. The interface temperature was 250 °C, and the ion source temperature was 200 °C. Helium was used as carrier gas at a flow rate of 1 mL/min. An electron beam of 70 eV was used to achieve ionization in full-scan mode (*m/z* 35–800). After a 6-min solvent delay time, the mass of AA was identified under 0.9 kV of detector voltage compared with the reference AA (St. Louis, MO, USA) using a similar mass spectra and retention time.

### Determination of TNF-α and IL-1β levels in ipsilateral brain tissue using the enzyme-linked immunosorbent assay (ELISA)

The thawed brain samples were weighed, dissected and homogenized in 9 volumes (1:9, *w/v*) of ice-cold normal saline. The homogenates were centrifuged for 15 min (3,000 rpm, 4 °C). The supernatants were used to measure the contents of TNF-α and IL-1β according to the manufacturer’s instructions (CUSABIO, Wuhan, China). Tissue protein concentrations were measured using the Bradford method.

### Western blot analysis

An exact weight of 0.2 g brain tissue was immersed once in 0.1 M ice-cold phosphate-buffered saline (BPS). After air drying, the tissue was placed in 500 μl ice-cold RIPA lysis buffer with protease inhibitors to prepare the tissue homogenates. After 30 minutes on ice, the homogenates were centrifuged for 5 min (12,000 rpm, 4 °C), and the supernatants were collected to be used as sample solutions for western blot analysis, as described in a previous study^31^. Adequate sample solutions were removed to measure the total protein concentrations using the bicinchoninic acid (BCA) method. The membranes were incubated with the following primary antibodies: AKT (1:2,000; Cell Signaling Technology, Boston), phospho-AKT (1:2,000; Cell Signaling Technology, Boston), mTOR (1:1,000; Cell Signaling Technology, Boston), phospho-mTOR (1:100; Cell Signaling Technology, Boston), phospho-p70S6K (1:200; Santa Cruz Biotechnology, California), p70S6K (1:1,000; Cell Signaling Technology, Boston) and β-actin (1:4,000; Santa Cruz Biotechnology, California). The band density was quantified using Image J software. The amount of protein expression is presented relative to the levels of β-actin.

### Assessment of neurological injury

The mNSS was performed as described in a previous study^60^. The mNSS includes a series of composite tests to evaluate the motor (muscle status, abnormal movement), sensory (visual, tactile, and proprioceptive), and reflex abilities using a set of test items. One point is awarded for failure to perform a particular task or for the absence of a tested reflex. Thus, a higher score indicates a more severe injury (normal score: 0; maximal deficit score: 18). Post-TBI, the mNSS was evaluated on the 1^st^, 3^rd^, 7^th^, 14^th^ and 21^st^ days to determine the severity of the injury.

### Cognitive assessment

Cognitive testing was conducted in a MWM as described previously^29^. Briefly, the rats were placed into the tank abutting the wall from one of the equivalently spaced starting locations, which were stochastically changed in every trial. On the 21^st^ day, a spatial probe trial was performed to assess the strength of the spatial memory retention by removing the platform. To achieve this goal, the rats were placed in the same start location facing the wall and then allowed to swim freely for 60 seconds in the pool without a platform to calculate the time spent in the target quadrant zone, in which the platform was positioned during acquisition trails, thereby indicating the degree of memory consolidation that occurred after learning. Behavioral parameters were tracked and analyzed using the ANY-maze video tracking system (Stoelting Co., USA).

### Statistical Analysis

All of the data are presented as the mean ± SEM. Statistical analyses were conducted using the SPSS 11.0 software package or Prism 5.0 (GraphPad) or SAS software. A repeated-measures ANOVA (RM ANOVA) was employed for mNSS. The RM ANOVA mixed model and two-factor RM ANOVA (group×time) were used for the statistical analysis of MWM scores. The remaining biochemical data were analyzed by two-way ANOVA. Statistical significance was defined as p < 0.05.

## Additional Information

**How to cite this article**: Xing, Z. *et al*. Xuefu Zhuyu decoction, a traditional Chinese medicine, provides neuroprotection in a rat model of traumatic brain injury via an anti-inflammatory pathway. *Sci. Rep.* **6**, 20040; doi: 10.1038/srep20040 (2016).
